# Delayed Laparoscopic Cholecystectomy With Fluorescent Cholangiography for Acute Cholecystitis: Is It Safe?

**DOI:** 10.1111/ases.70092

**Published:** 2025-06-03

**Authors:** Tsuyoshi Igami, Takuya Ishikawa, Kentaro Yamao, Yasuyuki Mizutani, Yukihiro Yokoyama, Takashi Mizuno, Junpei Yamaguchi, Shunsuke Onoe, Masaki Sunagawa, Nobuyuki Watanabe, Shoji Kawakatsu, Hiroki Kawashima, Tomoki Ebata

**Affiliations:** ^1^ Division of Surgical Oncology, Department of Surgery Nagoya University Graduate School of Medicine Nagoya Japan; ^2^ Department of Gastroenterology and Hepatology Nagoya University Graduate School of Medicine Nagoya Japan

**Keywords:** acute cholecystitis, delayed laparoscopic cholecystectomy, fluorescent cholangiography, percutaneous transhepatic gallbladder drainage, single‐incision laparoscopic cholecystectomy

## Abstract

**Background:**

According to the Tokyo Guidelines 2018 (TG‐18), delayed laparoscopic cholecystectomy (DLC) after recovering from acute cholecystitis (AC) is recommended for patients with poor status. Moreover, DLC for patients with good status remains controversial, and TG‐18 does not include clinical questions regarding fluorescent cholangiography (FC). In this study, we evaluated the clinical value and safety of FC during DLC.

**Methods:**

We performed DLC in 226 patients after recovering from AC. The electronic medical records of these patients were retrospectively reviewed, focusing on preoperative assessment and intraoperative and postoperative outcomes. Biliary and/or arterial injuries were treated as intraoperative complications.

**Results:**

Of the study patients, 144 underwent DLC with FC. Among the remaining 82 patients who underwent DLC without FC, the rate of intraoperative complications was 7.3% (*n* = 6), which was significantly higher than in those who underwent DLC with FC (0%) (*p* = 0.002). The rate of conversion to open cholecystectomy during DLC with FC (1.4%) was significantly lower than that during DLC without FC (15.9%). The mean operative time was not significantly different between the patients who underwent DLC with and without FC (*p* = 0.503). The mean blood loss and postoperative complications in patients who underwent DLC with FC were significantly lower than those who underwent DLC without FC (*p* = 0.041 and *p* = 0.002, respectively).

**Conclusions:**

Utilizing FC can reduce intraoperative and postoperative complications, the conversion rate, and blood loss during DLC; therefore, DLC with FC is recognized as a safe procedure for patients with AC.

## 1 Introduction

According to the Tokyo Guidelines 2018 (TG‐18) [1], it was recommended that cholecystectomy, including laparoscopic cholecystectomy (LC), for acute cholecystitis (AC) of any grade should be performed as soon as possible after the diagnosis of AC; however, the actual status of AC is quite complex because of the concomitance of common bile duct (CBD) stones and/or cholangitis. Additionally, comorbidities and/or the administration of a drug that requires an adequate cessation period are important for deciding indications for urgent surgery, and it is recommended that the preoperative status, including such a situation, is assessed according to both the American Society of Anesthesiologists physical status (ASA‐PS) and the Charlson comorbidity index (CCI) in TG‐18 [1, 2, 3, 4]. Accordingly, delayed surgery, including delayed LC (DLC), after recovering AC is recommended for patients with poor status [1]. Moreover, implementing DLC for patients with good status remains controversial. In addition, reports regarding the clinical value of fluorescent cholangiography (FC) during LC for cholecystitis [5, 6, 7, 8, 9, 10, 11, 12, 13]; however, TG‐18 does not include clinical questions regarding FC. In this study, we evaluated the clinical value and safety of DLC with FC for patients with AC.

## 2 Materials and Methods

### 2.1 Patients

We performed LC for AC in 229 patients at Nagoya University Hospital between July 2010 and December 2023. Among them, only three patients underwent early LC, which occurred within 72 h after the diagnosis of AC, because we basically selected DLC for the treatment of AC. The remaining 226 patients underwent DLC, which involved LC more than 72 h after the diagnosis of AC, and their electronic medical records were retrospectively reviewed, focusing on preoperative assessment and intraoperative and postoperative outcomes. Biliary and/or arterial injuries during DLC were treated as intraoperative complications.

This study was approved by the Human Research Review Committee of Nagoya University Hospital (approval number 2023‐0353). Because this study is a retrospective study, instead of individual informed consent, all patients were guaranteed the opportunity to refuse according to the information disclosure of this study on the webpage (https://nagoya.bvtis.com/rinri/publish.aspx).

### 2.2 Diagnosis and Treatment of AC

All patients with AC underwent blood examination and computed tomography, and the severity of AC was classified according to TG‐18 [14]. In addition, patient status was classified according to TG‐18, that is, either ASA‐PS ≥ 3 or CCI ≥ 6 was treated as poor physical status to perform urgent surgery for mild (Grade I) and moderate (Grade II) AC. Patients with either an ASA‐PS ≥ 3 or a CCI ≥ 4 were treated as having poor physical status to perform urgent surgery for severe (Grade III) AC [1]. Blood culture was performed in patients whose body temperature was > 38.0°C. The antibiotics recommended by our infection control teams were immediately administered [15].

When CBD stones and/or cholangitis are confirmed and/or suspected, endoscopic sphincterotomy (EST), endoscopic papillary balloon dilatation (EPBD), endoscopic nasobiliary drainage (ENBD), and/or endoscopic biliary stenting (EBS) are appropriately performed according to the severity of cholangitis of TG‐18 [16, 17, 18]. At the time of these treatments, bile culture was performed.

Percutaneous transhepatic gallbladder drainage (PTGBD) was selected for the following patients: patients with severe comorbidities, patients receiving anticoagulant and/or antithrombotic drugs, patients confirmed and/or suspected of having gangrenous cholecystitis and/or abscess around the gallbladder (GB), and/or patients without improvement of symptoms with AC despite the administration of antibiotics [19]. For the PTGBD procedure, a pig‐tail catheter was inserted via the transhepatic route into the GB under the guidance of ultrasonography, and the location of the catheter tip was checked under radiographic guidance. At the time of PTGBD, bile culture was performed.

Among patients whose bacteria were confirmed by blood and/or bile cultures, antibiotics sensitive to the detected bacterial species were appropriately selected. The administration of antibiotics was continued until all of the following conditions were confirmed: improvement of symptoms, decrease in body temperature < 37.0°C, normalization of white blood cell (WBC) counts, and decrease in C‐reactive protein (CRP) < 3.00 mg/dL.

During the abovementioned treatment, with the assessment of severe comorbidity and/or securement of appropriate cessation periods of anticoagulant and/or antiplatelet drugs, cholecystectomy was scheduled according to the patients' requests after recovery from AC, that is, as soon as possible, during the next vacation, until the baby became slightly older and/or finished the ongoing work.

### 2.3 Surgical Procedure and FC

**FIGURE 1:**
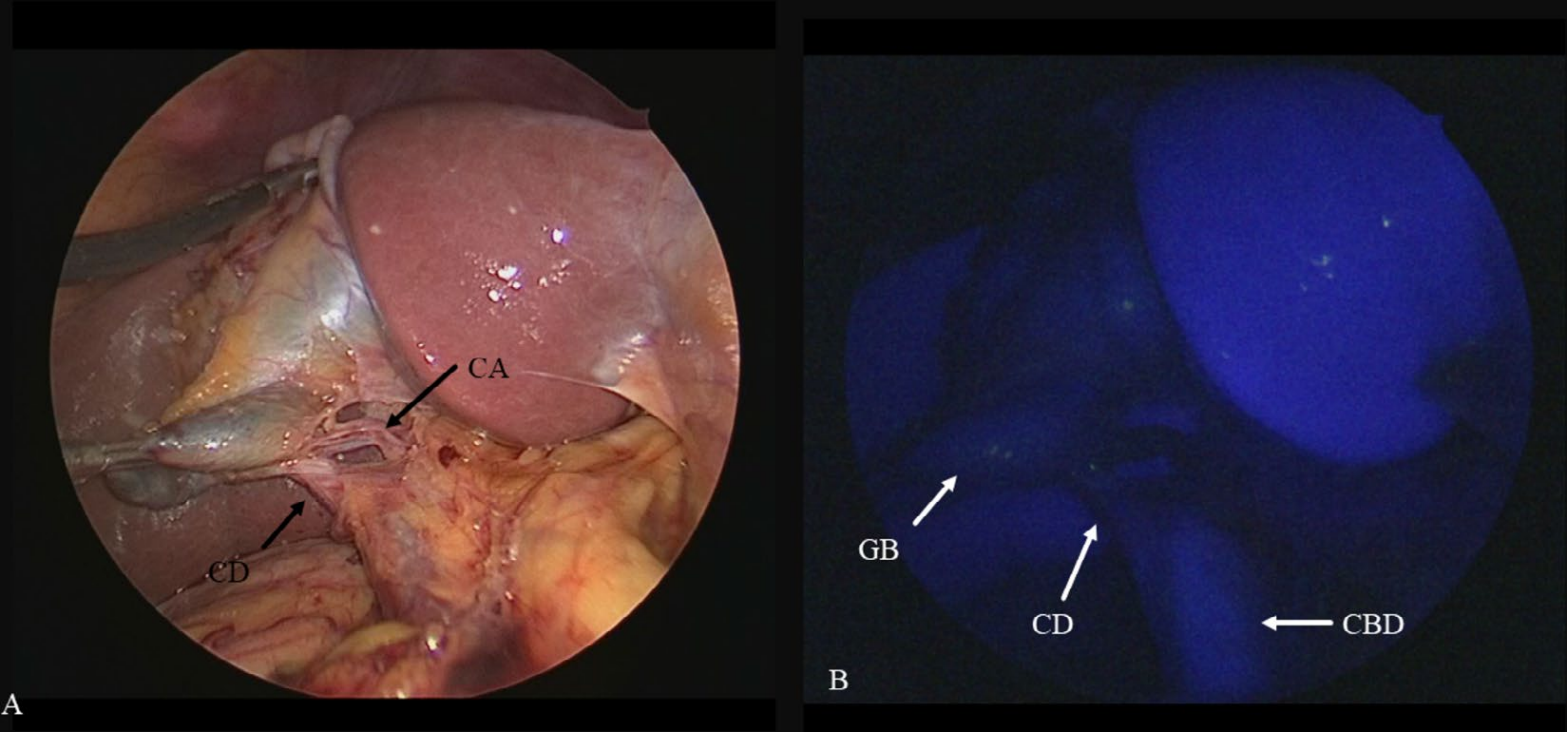
Intraoperative findings of the “critical view of safety.” (A) Normal laparoscopic view after dissection of Calot's triangle showing that the cystic duct (CD) and the cystic artery (CA) were exposed, resulting in a “critical view of safety.” (B) Fluorescent cholangiography showing the CD, the common bile duct (CBD), and the gallbladder (GB) is well visualized.

**FIGURE 2:**
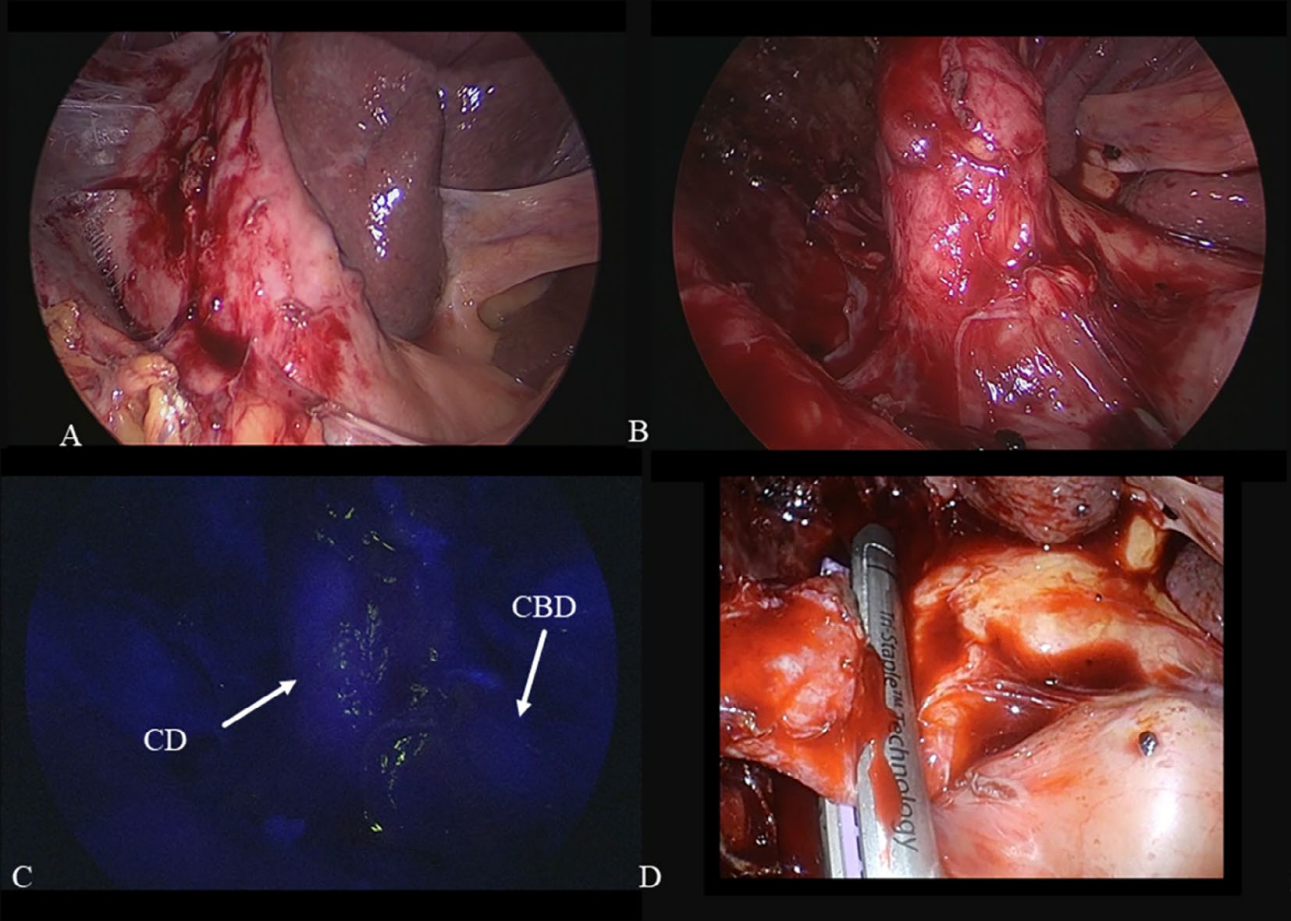
Intraoperative findings of the fundus‐down procedure. (A) Normal laparoscopic view before dissection of Calot's triangle revealed that dissection of Calot's triangle could not be performed due to severe inflammation around the gallbladder neck, but the connective tissue around the gallbladder fundus was mildly inflamed; therefore, the fundus‐down approach was selected. (B) Normal laparoscopic view after the fundus‐down approach illustrates that the cystic artery cannot be exposed from the connective tissue around the cystic duct (CD). (C) Fluorescent cholangiography after the fundus‐down approach shows that the CD and the common bile duct (CBD) are well visualized. (D) The CD and the cystic artery were divided together via an endoscopic linear stapler.

**FIGURE 3:**
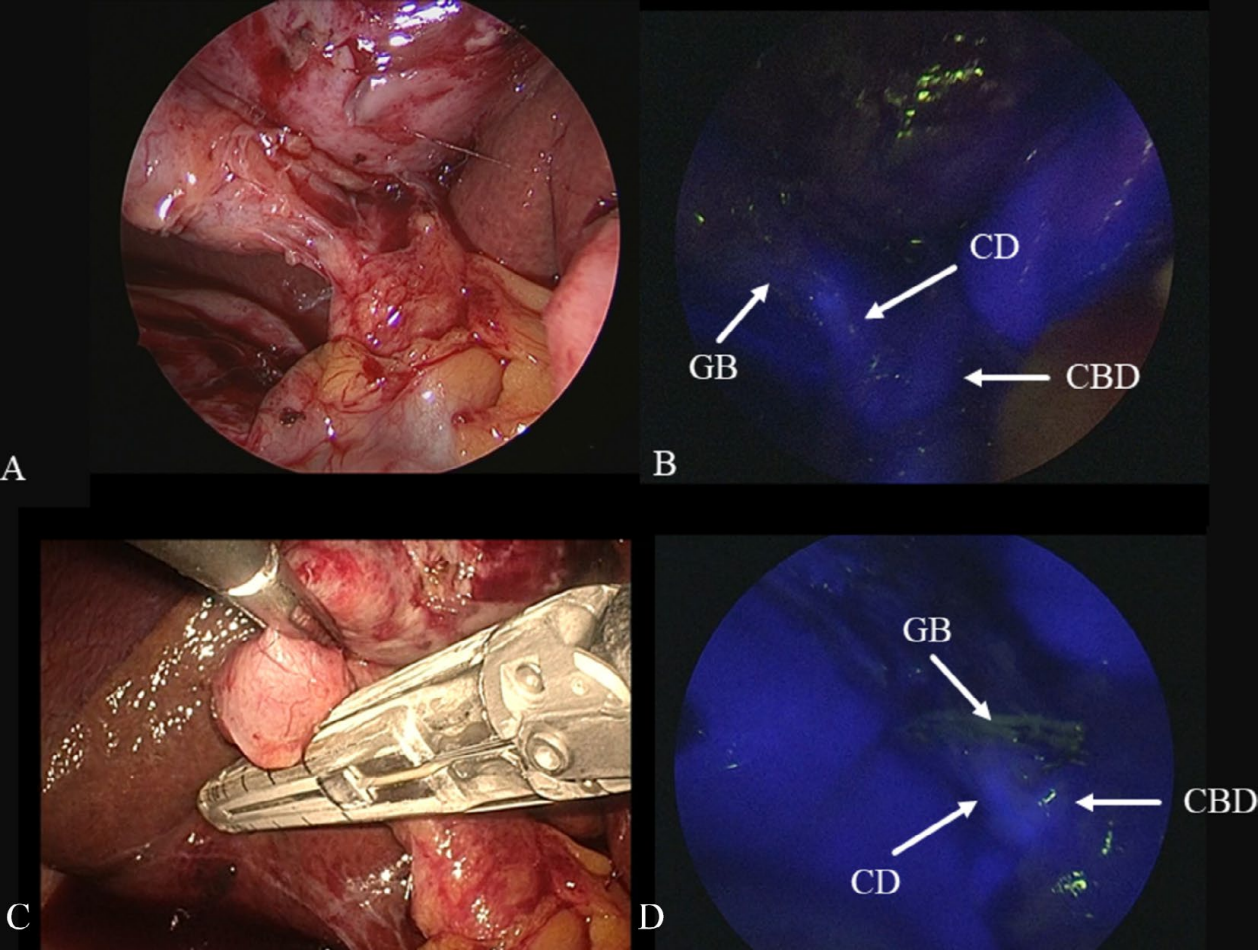
Intraoperative findings of subtotal cholecystectomy. (A) Normal laparoscopic view during dissection of Calot's triangle showing that the critical view of safety could not be achieved due to severe inflammation in Calot's triangle. (B) Fluorescent cholangiography depicting the cystic duct (CD), the common bile duct (CBD), and the gallbladder (GB) neck. (C) The GB neck was divided via an endoscopic linear stapler. (D) The staple line was at the stump of the GB neck, with visualization of the CD and the CBD under fluorescent cholangiography.

Patients with one and multiple upper abdominal laparotomies underwent conventional LC (CLC) and open cholecystectomy (OC), respectively. For patients without a history of upper abdominal laparotomy, single‐incision LC (SILC) was performed according to our previous reports [20, 21, 22, 23, 24, 25, 26, 27]. During LC, dissection of Calot's triangle was performed according to the “critical view of safety” (CVS) concept [28] (Figure 1). When CVS was unobtainable because of severe inflammation around the cystic duct (CD) or the cystic artery (CA), the fundus‐down approach (FDA) (Figure 2) and/or subtotal cholecystectomy (STC) (Figure 3) were appropriately selected as conventional bailout procedures [29].

**FIGURE 4:**
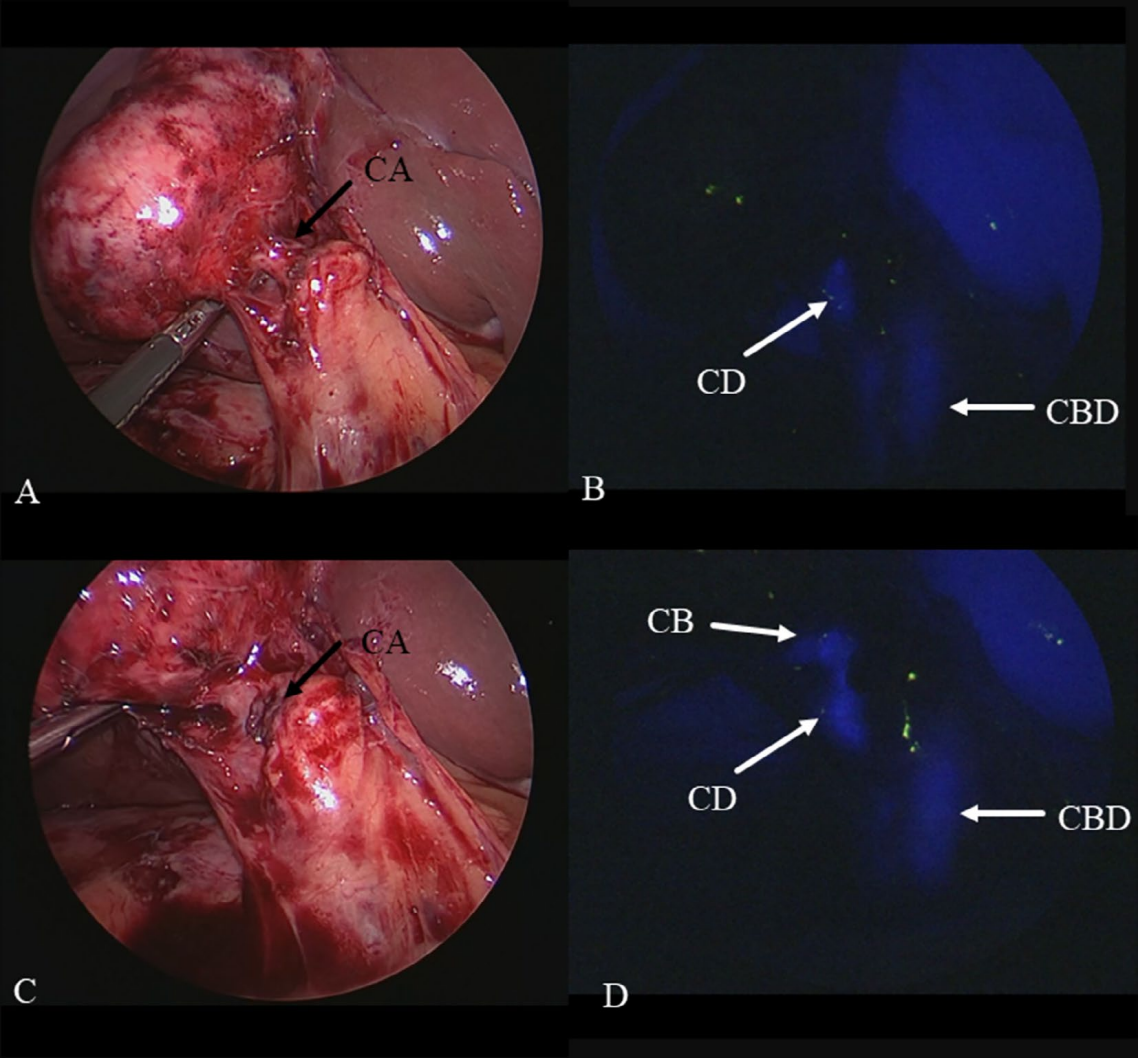
Intraoperative findings of the previous division of the cystic artery (CA) before achieving the critical view of safety. (A) Normal laparoscopic view showing that the CA could be exposed, but dissection of Calot's triangle could not be continued due to severe inflammation in Calot's triangle. (B) Fluorescent cholangiography revealed that the running courses of the cystic duct (CD) and the common bile duct (CBD) were well visualized, but the confluence of the CD and the gallbladder (GB) could not be visualized because of the CA. (C) The CA was divided using a 5‐mm clip. (D) The confluence of the CD and the GB was well visualized after the previous division of the CA under the guidance of fluorescent cholangiography.

**FIGURE 5:**
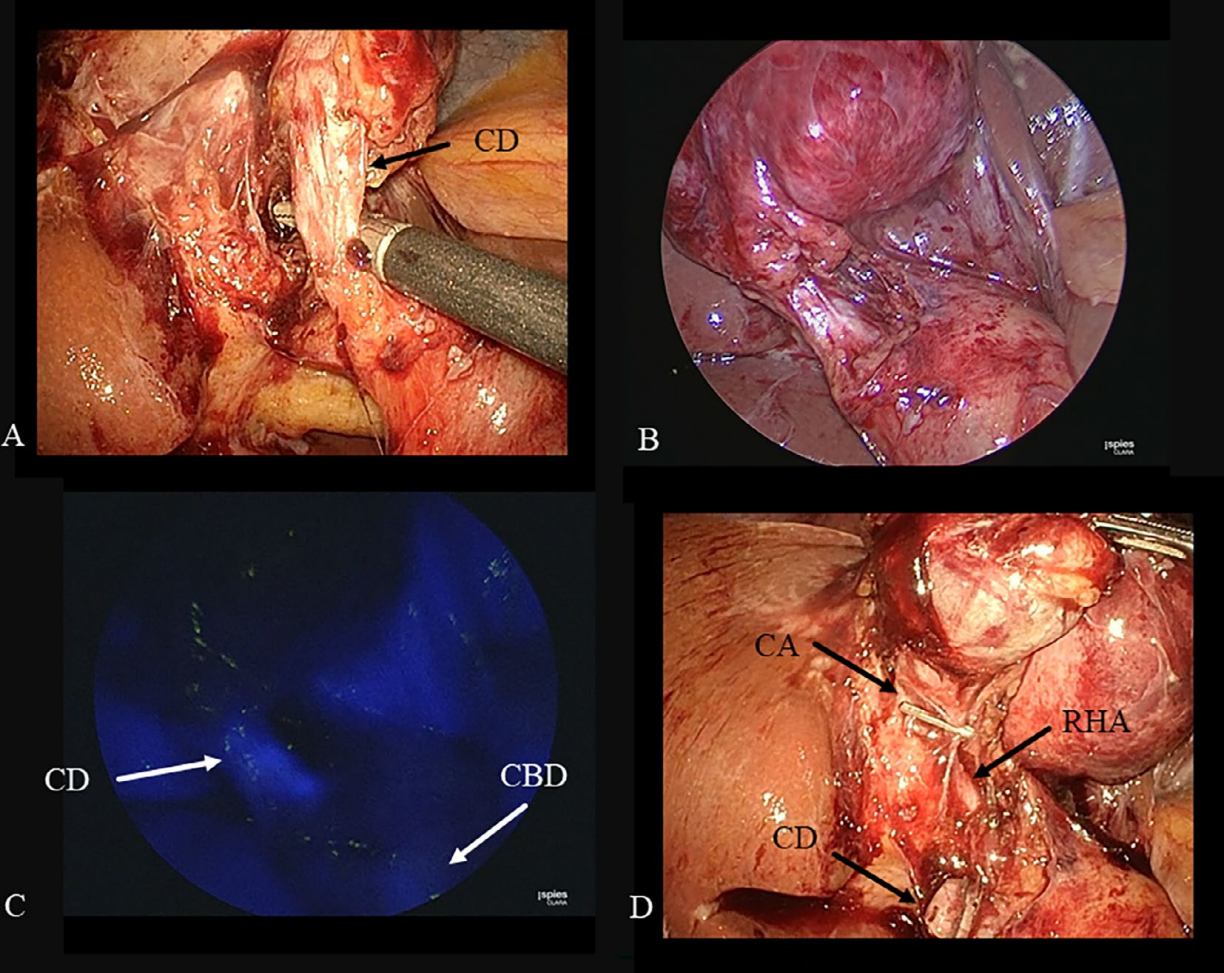
Intraoperative findings of previous division of the cystic duct (CD) before achieving the critical view of safety. (A) Normal laparoscopic view during dissection of Calot's triangle revealed that the CD could be exposed, but the connective tissues at the caudal side of the gallbladder neck could not be dissected due to severe inflammation. (B) Normal laparoscopic view showing that the cystic artery (CA) could not be found at the cranial side of the gallbladder neck. (C) Fluorescent cholangiography showed that the CD and the common bile duct (CBD) were well visualized. (D) The CA could be clipped with no injury to the right hepatic artery (RHA) in the connective tissue at the caudal side of the gallbladder neck after the previous division of the CD under the guidance of fluorescent cholangiography.

Based on our previous report [23, 24, 25, 26, 27], FC was performed and was aggressively utilized during LC for patients with AC. As a fluorescent source, 2.5 mg of indocyanine green (Diagnogreen; Daiichi Sankyo Co., Tokyo, Japan) was intravenously injected after endotracheal intubation of the patient in the operating room. In patients whose CD was detected during dissection of Calot's triangle under FC, when severe inflammation obstructed CVS, previous division of CA before obtaining CVS (PDCA) under the guidance of FC (Figure 4) and previous division of CD before obtaining the CVS (PDCD) under the guidance of FC (Figure 5) were treated as distinctive bailout procedures with FC.

### 2.4 Statistical Analysis

Continuous variables, expressed as the mean ± standard deviation (SD) unless specified otherwise, were compared via the Mann–Whitney *U*‐test. Categorical variables were analyzed using the chi‐square test or Fisher's exact test, as appropriate. All tests were two‐sided, and *p* < 0.05 was considered statistically significant. Statistical calculations were performed with SPSS version 29 (IBM).

## 3 Results

### 3.1 Clinical Characteristics of the Study Patients

Among the 226 study patients, 150 were male and 76 were female, with a mean age of 65 ± 14 (range: 21–92) years. Among them, 88, 133, and 5 patients were diagnosed with Grades I, II, and III AC, respectively. Additionally, 122 (54.0%) patients were simultaneously confirmed and/or suspected of having CBD stones and/or cholangitis and were aggressively selected for treatment of CBD stones and/or cholangitis rather than urgent surgery. Regarding the procedures used for the treatment of CBD stones and/or cholangitis, which included overlap, 85, 13, 22, and 34 patients underwent EST, EPBD, ENBD, and EBS, respectively. According to preoperative assessments via the ASA‐PS and CCI, 43, 70, and 5 patients were evaluated for poor physical status to perform urgent surgery for Grades I, II, and III AC, respectively. Regarding the administration of drugs requiring appropriate cessation periods before surgery, which included overlap, 58, 5, 2, and 1 patients were administered antithrombotic drugs (anticoagulant and/or antiplatelet drugs), sodium‐glucose cotransporter 2 inhibitors, osteoporosis treatment drugs, and female hormones, respectively.

Excluding the patients with selection of treatment of CBD stones and/or cholangitis, poor physical status, and/or administration of drugs requiring appropriate cessation periods before surgery from the 226 study patients, there were 53 (23.4%) patients with a potential status to perform urgent surgery for AC.

### 3.2 Comparison Between Patients Who Underwent DLC With and Without Utilization of FC

Regarding the surgical procedure, CLC and SILC were performed in 62 and 164 patients, respectively. The 13 patients who underwent CLC and the 2 patients who underwent SILC required conversion to OC, and the other 2 patients were converted from SILC to CLC. Accordingly, 211 (93.4%) patients achieved DLC. Among the 138 patients with Grades II/III AC, 86 (62.3%) (Grades II/III, 81/5) patients underwent PTGBD preoperatively.

Among the 226 study patients, 144 (63.7%) patients underwent DLC with FC, and there were 90 males and 54 females, with a mean age of 65 ± 15 years and a mean body mass index (BMI) of 23.7 ± 4.4 kg/m^2^. The remaining 82 patients underwent DLC without FC, and there were 60 males and 22 females, with a mean age of 65 ± 12 years and a mean BMI of 23.6 ± 3.6 kg/m^2^. The ratio of males to females, the mean age, and the mean BMI were not significantly different between the two patient groups (*p* = 0.110, *p* = 0.589, and *p* = 0.968, respectively). These trends were recognized among the patient groups according to the severity of AC.

Comparisons between the patients who underwent DLC with and without FC are shown in Table 1. The rate of conventional bailout procedures was not significantly different between DLC with and without FC (7.6% vs. 15.9%, *p* = 0.072); however, in patients with Grade II AC without PTGBD, that during DLC with FC (3.4%) was significantly lower than that during DLC without FC (26.1%) (*p* = 0.035). Among the eight patients who underwent FDA without FC, there were injuries to the right hepatic duct (RHD) and the right hepatic artery (RHA) in each patient. The rate of distinctive bailout procedures was significantly greater during DLC with FC (29.9%) than during DLC without FC (17.1%) (*p* = 0.039), and this difference was remarkable in patients with Grade II AC requiring PTGBD (28.8% vs. 7.4%, *p* = 0.028). PDCA and PDCD with FC were performed in 36 and 7 patients, respectively, with no intraoperative complications. Moreover, PDCA and PDCD without FC were performed in 13 and 1 patient, respectively. Among the four patients who experienced intraoperative complications during PDCA without FC, two patients experienced injury to CD, and each patient experienced injury to CBD or CA. The rate of intraoperative complications during PDCA without FC (30.8%) was significantly greater than that with FC (0%) (*p* = 0.003). Fortunately, the patient who underwent PDCD without FC had no intraoperative complications.

**TABLE 1:** Comparison between the patients who underwent delayed laparoscopic cholecystectomy with and without utilization of fluorescent cholangiography.

	Total			Grade I			Grade II without PTGBD			Grades II and III with PTGBD		
	FC (+)	FC (−)	*p*	FC (+)	FC (−)	*p*	FC (+)	FC (−)	*p*	FC (+)	FC (−)	*p*
	*n* = 144	*n* = 82		*n* = 56	*n* = 32		*n* = 29	*n* = 23		*n* = 59	*n* = 27	
Conventional bailout procedures	11	13	0.072	1	2	0.551	1	6	0.035	9	5	0.757
FDA	8	8		1	1		0	4		7	3	
STC	3	5		0	1		1	2		2	2	
Distinctive bailout procedures	43	14	0.039	18	9	0.812	8	3	0.308	17	2	0.028
PDCA^a^	36	13		15	9		7	3		14	1	
PDCD^a^	7	1		3	0		1	0		3	1	
Intraoperative complications	0	6	0.002	0	0	> 0.999	0	5	0.013	0	1	0.314
Injury of the common bile duct	0	1		0	0		0	1		0	0	
Injury of the right hepatic duct	0	1		0	0		0	0		0	1	
Injury of the right hepatic artery	0	1		0	0		0	1		0	0	
Injury of the cystic duct	0	2		0	0		0	2		0	0	
Injury of the cystic artery	0	1		0	0		0	1		0	0	
Conversion to OC	2	13	< 0.001	0	2	0.130	1	8	0.007	1	3	0.090
Operative time (min)^b^	123 ± 47	117 ± 43	0.503	100 ± 27	107 ± 42	0.596	139 ± 56	127 ± 43	0.549	138 ± 47	123 ± 41	0.234
Blood loss (mL)^b^	13 ± 30	25 ± 62	0.041	5 ± 13	19 ± 72	0.074	25 ± 45	36 ± 59	0.327	16 ± 29	24 ± 47	0.374
Postoperative complications^b^	4	8	0.002	1	1	> 0.999	2	3	0.324	1	4	0.024
Common bile duct stones	0	6		0	1		0	2		0	3	
Pancreatitis	1	0		1	0		0	0		0	0	
Appendicitis	1	0		0	0		0	0		1	0	
Aspiration pneumonia	1	0		0	0		1	0		0	0	
Urethral injury	1	1		0	0		1	0		0	1	
Intraabdominal bleeding	0	1		0	0		0	1		0	0	

Abbreviations: FC, fluorescent cholangiography; FDA, fundus down approach; OC, open cholecystectomy; PDCA, previous division of the cystic artery under FC before achievement of critical view of safety; PDCD, previous division of the cystic duct under FC before achievement of critical view of safety; PTGBD, percutaneous transhepatic gallbladder drainage; STC, subtotal cholecystectomy.

^a^ In the FC(−) group, the procedures were performed without a guidance of FC.

^b^ Excluding the patients who required conversion to OC.

Both the rates of intraoperative complications and conversion to OC during DLC with FC (0.0% and 1.4%) were significantly lower than those during DLC without FC (7.3% and 15.9%) (*p* = 0.002 and *p* < 0.001), and these differences were significant among patients with Grade II AC without requiring PTGBD (0.0% vs. 21.7%, *p* = 0.013, and 3.4% vs. 34.5%, *p* = 0.007). Among the 15 patients who underwent conversion to OC, 11 patients had unrelated reasons for intraoperative complications, that is, severe adhesion due to previous surgery in 5 patients, severe adhesion due to severe inflammation around the GB in 5 patients, and irremovable stones due to Mirizzi syndrome in one patient. Among the remaining four patients who underwent conversion to OC, the reasons for intraoperative complications included injury to CD, CBD, RHD, and RHA. The remaining two patients with intraoperative complications achieved DLC with repair of the damaged cystic CD or CA.

Among the patients who achieved DLC, the mean operative time was not significantly different between DLC with and without FC (123 ± 47 vs. 117 ± 43 min, *p* = 0.503), and this trend was recognized despite the severity of AC. Overall, the mean blood loss during DLC with FC (13 ± 30 mL) was significantly lower than that during DLC without FC (25 ± 62 mL) (*p* = 0.041), but this difference was not recognized among the patient groups according to the severity of AC. Postoperative complications after DLC with FC (2.8%) were significantly lower than those after DLC without FC (9.8%) (*p* = 0.002), and this difference was remarkable in patients with Grade II AC requiring PTGBD (1.7% vs. 14.8%, *p* = 0.024).

Among the 53 patients with a potential status of urgent surgery for AC, 33 and 20 patients underwent DLC with and without FC, respectively. Among these two patient groups, the ratio of males to females (16:17 vs. 14:6), the mean age (55 ± 14 vs. 56 ± 8 years), and the mean BMI (25.1 ± 5.0 vs. 25.4 ± 3.7 kg/m^2^) were not significantly different (*p* = 0.159, *p* = 0.849, and *p* = 0.646), and there were no intraoperative complications. In the former patient group, one patient required conversion to OC for Mirizzi syndrome. Among the patients who achieved DLC with FC (*n* = 32) and without FC (*n* = 20), the mean operative time (127 ± 50 vs. 105 ± 37 min) and mean blood loss (8 ± 19 vs. 14 ± 34 mL) were not significantly different (*p* = 0.174 and *p* = 0.976). Fortunately, there were no postoperative complications.

### 3.3 Detectability of Biliary Structures Before Dissection of Calot's Triangle Under FC

The detectabilities of biliary structures before dissection of Calot's triangle in the 142 patients who achieved DLC with FC are shown in Table 2. The detectabilities of CD, CBD, and GB before dissection of Calot's triangle were 49.3% (*n* = 70), 85.9% (*n* = 122), and 53.5% (*n* = 76), respectively. The percentage of patients whose biliary structures were not detected before dissection of Calot's triangle was 5.6% (*n* = 8).

**TABLE 2:** Detectabilities of the biliary structures before dissection of the Calot's triangle of the 142 patients who underwent delayed laparoscopic cholecystectomy with fluorescent cholangiography.

	Cystic duct		Common bile duct		Gallbladder		Any biliary structures	
	49.3% (*n* = 70)	*p*	85.9% (*n* = 122)	*p*	53.5% (*n* = 76)	*p*	94.4% (*n* = 134)	*p*
Body mass index								
< 25.0 kg/m^2^ (*n* = 100)	57.0% (*n* = 57)	0.006	88.0% (*n* = 88)	0.296	49.0% (*n* = 49)	0.102	94.0% (*n* = 94)	> 0.999
≥ 25.0 kg/m^2^ (*n* = 42)	31.0% (*n* = 13)		81.0% (*n* = 34)		64.3% (*n* = 27)		95.2% (*n* = 40)	
ICG‐FC^a^								
< 44 min (*n* = 72)	55.6% (*n* = 40)	0.136	91.7% (*n* = 66)	0.055	51.4% (*n* = 37)	0.618	94.3% (*n* = 66)	> 0.999
≥ 44 min (*n* = 70)	42.9% (*n* = 30)		80.0% (*n* = 56)		55.7% (*n* = 39)		94.4% (*n* = 68)	
Grade^b^								
I (*n* = 56)	51.8% (*n* = 29)	0.732	92.9% (*n* = 52)	0.082	67.9% (*n* = 38)	0.006	98.2% (*n* = 55)	0.147
II/III (*n* = 86)	47.7% (*n* = 41)		81.4% (*n* = 70)		44.2% (*n* = 38)		91.9% (*n* = 79)	
Max‐WBC^c^								
< 13 000 counts (*n* = 79)	51.9% (*n* = 41)	0.504	88.6% (*n* = 70)	0.338	54.4% (*n* = 43)	0.866	93.7% (*n* = 74)	> 0.999
≥ 13 000 counts (*n* = 63)	46.0% (*n* = 29)		82.5% (*n* = 52)		52.4% (*n* = 33)		95.2% (*n* = 60)	
Max‐CRP^d^								
< 13.98 mg/dL (*n* = 79)	51.9% (*n* = 41)	0.504	94.9% (*n* = 75)	0.001	62.0% (*n* = 49)	0.028	97.5% (*n* = 77)	0.139
≥ 13.98 mg/dL (*n* = 63)	46.0% (*n* = 29)		74.6% (*n* = 47)		42.9% (*n* = 27)		90.5% (*n* = 57)	
PTGBD								
− (*n* = 84)	46.3% (*n* = 39)	0.495	88.1% (*n* = 74)	> 0.999	58.3% (*n* = 49)	0.176	94.1% (*n* = 79)	> 0.999
+ (*n* = 58)	53.5% (*n* = 31)		87.9% (*n* = 51)		46.6% (*n* = 27)		94.8% (*n* = 55)	
EST/EPBD								
− (*n* = 85)	52.9% (*n* = 45)	0.309	89.4% (*n* = 76)	0.602	54.1% (*n* = 46)	0.866	94.1% (*n* = 80)	> 0.999
+ (*n* = 57)	43.9% (*n* = 25)		86.0% (*n* = 49)		52.6% (*n* = 30)		94.7% (*n* = 54)	
ENBD/EST								
− (*n* = 109)	53.2% (*n* = 58)	0.113	90.8% (*n* = 99)	0.073	55.1% (*n* = 60)	0.554	97.3% (*n* = 106)	0.017
+ (*n* = 33)	36.4% (*n* = 12)		78.8% (*n* = 26)		48.5% (*n* = 16)		84.9% (*n* = 28)	

Abbreviations: EBS, endoscopic biliary stenting; ENBD, endoscopic nasobiliary drainage; EPBD, endoscopic papillary balloon dilatation; EST, endoscopic sphincterotomy; PTGBD, percutaneous transhepatic gallbladder drainage.

^a^ Interval between injection of ICG and start of fluorescent cholangiography.

^b^ Grade of acute cholecystitis.

^c^ The maximum of white blood cell counts between diagnosis of acute cholecystitis and surgery.

^d^ The maximum of C‐reactive protein between diagnosis of acute cholecystitis and surgery.

The detectability of CD before dissection of Calot's triangle in patients with a BMI ≥ 25.0 kg/m^2^ (31.0%) was significantly lower than those with a BMI < 25.0 kg/m^2^ (57.0%) (*p* = 0.006). Among these two patient groups, the detectability rates of CBD (81.0% vs. 88.0%) and GB (64.3% vs. 49.0%) were not significantly different (*p* = 0.296 and *p* = 0.102, respectively). Additionally, the detectability of any biliary structures before dissection of Calot's triangle was not significantly different between these two patient groups (95.2% vs. 94.0%, *p* > 0.999).

The mean interval between the injection of ICG and the start of FC was 44 ± 14 min (range: 10–115 min). The detectabilities of CD (42.9% vs. 55.6%), CBD (80.0% vs. 91.7%), GB (55.7% vs. 51.4%), and any biliary structures (94.4% vs. 94.3%) before dissection of Calot's triangle were not significantly different (*p* = 0.136, *p* = 0.055, *p* = 0.618, and *p* > 0.999).

The detectability of GB before dissection of Calot's triangle in patients with Grade I AC (67.9%) was significantly greater than those with Grades II/III AC (44.2%) (*p* = 0.006). Among these two patient groups, the detectabilities of CD (51.8% vs. 47.7%), CBD (92.9% vs. 81.4%), and any biliary structures (98.2% vs. 91.9%) before dissection of Calot's triangle were not significantly different (*p* = 0.732, *p* = 0.082, and *p* = 0.147, respectively). The mean values of the maximum WBC count (Max‐WBC) and the maximum CRP level (Max‐CRP) between the diagnosis of AC and surgery were 13 000 ± 5100 counts (range: 2500–33 800 counts) and 13.98 ± 11.44 mg/dL (range: 0.06–53.56 mg/dL), respectively. Among the patients with Max‐WBC < 13 000 and ≥ 13 000 counts, the detectabilities of CD (51.9% vs. 46.0%), CBD (88.6% vs. 82.5%), GB (54.4% vs. 52.4%), and any biliary structures (93.7% vs. 95.2%) were not significantly different (*p* = 0.504, *p* = 0.338, *p* = 0.866, and *p* > 0.999). The detectabilities of CBD and GB in patients with a Max‐CRP < 13.98 mg/dL (94.9% and 62.0%) were significantly greater than those in patients with a Max‐CRP ≥ 13.98 mg/dL (74.6% and 42.9%), respectively (*p* = 0.001 and *p* = 0.028). Among these two patient groups, the detectabilities of CD (51.9% vs. 46.0%) and any biliary structures (97.5% vs. 90.5%) were not significantly different (*p* = 0.504 and *p* = 0.139).

Among the patients with and without preoperative PTGBD, the detectabilities of CD (53.5% vs. 46.3%), CBD (87.9% vs. 88.1%), GB (46.6% vs. 58.3%), and any biliary structures (94.8% vs. 94.1%) were not significantly different (*p* = 0.495, *p* > 0.999, *p* = 0.176, and *p* > 0.999), respectively. Among the patients with and without preoperative EST and/or EPBD, the detectabilities of CD (43.9% vs. 52.9%), CBD (86.0% vs. 89.4%), GB (52.6% vs. 54.1%), and any biliary structures (94.7% vs. 94.1%) were not significantly different (*p* = 0.309, *p* = 0.602, *p* = 0.866, and *p* > 0.999), respectively. The detectability of any biliary structures in the patients with preoperative ENBD and/or EBS (84.9%) was significantly lower than those without preoperative ENBD and/or EBS (97.3%) (*p* = 0.017). Among these two patient groups, the detectabilities of CD (36.4% vs. 53.2%), CBD (78.8% vs. 90.8%), and GB (48.5% vs. 55.1%) were not significantly different (*p* = 0.113, *p* = 0.073, and *p* = 0.554), respectively.

### 3.4 Detectability of Biliary Structures During Dissection of Calot's Triangle Under FC

The detectabilities of CD, CBD, and GB during dissection of Calot's triangle were 89.4% (*n* = 127), 95.1% (*n* = 135), and 54.2% (*n* = 77), respectively. The biliary structures of only one (0.7%) patient could not be detected during dissection of Calot's triangle. Among the 72 patients whose CD was undetectable before dissection of Calot's triangle under FC, 57 (79.2%) patients whose CD was changed to detectable during dissection of Calt's triangle. Similarly, among the 20 patients whose CBD was undetectable before dissection of Calot's triangle under FC, 13 (65.0%) patients whose CBD was changed to be detectable during dissection of Calot's triangle. Moreover, among the 66 patients whose GB was undetectable before dissection of Calot's triangle under FC, only 1 (1.5%) patient whose GB was changed to detectable during dissection of Calot's triangle.

**FIGURE 6:**
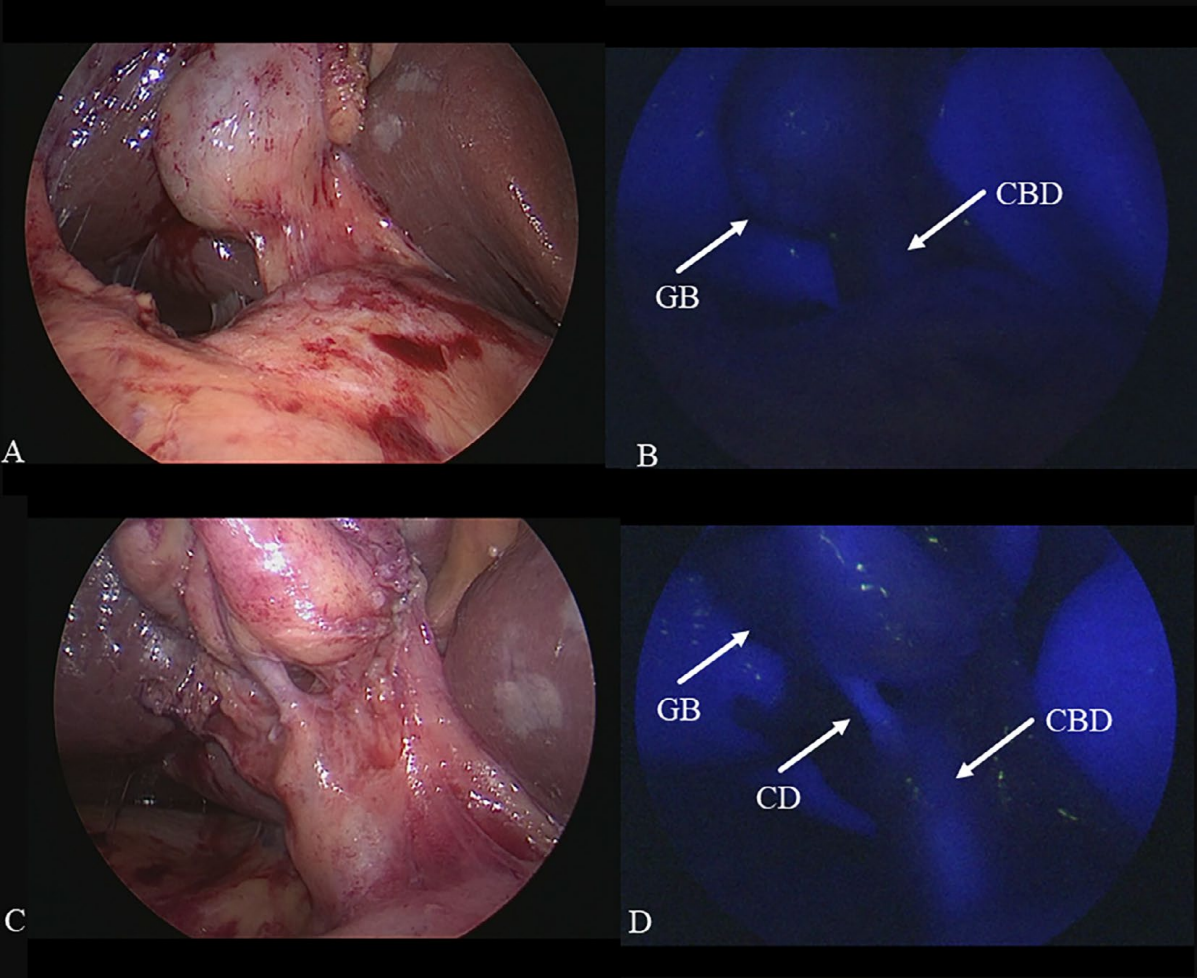
Change in the cystic duct (CD) from undetectable before dissection of Calot's triangle to detectable during dissection of Calot's triangle under fluorescent cholangiography. (A) Normal laparoscopic view showing severe inflammation in Calot's triangle. (B) Fluorescent cholangiography revealed that the gallbladder (GB) and the common bile duct (CBD) were well visualized, but the CD was undetectable. (C) Normal laparoscopic view showing that it was difficult to achieve a safe critical view. (D) Fluorescent cholangiography indicated that the CD was detectable with good visualization of the GB, and the CBD was visualized during the dissection of Calot's triangle.

A comparison of the patients with and without changes in CD from undetectable before dissection of Calot's triangle to detectable during dissection of Calot's triangle under FC (CHANGE‐IN‐CD) (Figure 6) is shown in Table 3. Regarding the rate of CHANGE‐IN‐CD, there were no significant differences among the patient groups according to BMI (*p* = 0.571), the interval between the injection of ICG and the start of FC (*p* = 0.381), or the grade of AC (*p* = 0.143). The rates of CHANGE‐IN‐CD in patients with a Max‐WBC ≥ 13 000 (64.7%) and in patients with a Max‐CRP ≥ 13.98 mg/dL (67.6%) were significantly lower than those in patients with a Max‐WBC < 13 000 (92.1%) and in patients with a Max‐CRP < 13.98 mg/dL (89.5%) (*p* = 0.008 and *p* = 0.040). Among the patients with and without preoperative PTGBD, preoperative EST and/or EPBD, and preoperative ENBD and/or EST, the rates of CHANGE‐IN‐CD (77.8% vs. 80.0%. 75.0% vs. 82.5%, and 71.4% vs. 82.4%) were not significantly different (*p* > 0.999, *p* = 0.562, and *p* = 0.346), respectively.

**TABLE 3:** Comparison of the patients with and without change in the cystic duct from undetectable before dissection of Calot's triangle to detectable during dissection of Calot's triangle under fluorescent cholangiography.

	Cystic duct (*n* = 72)		
	Change (+) (*n* = 57)	Change (−) (*n* = 15)	*p*
Body mass index			
< 25.0 kg/m^2^ (*n* = 43)	81.4% (*n* = 35)	18.6% (*n* = 8)	0.571
≥ 25.0 kg/m^2^ (*n* = 29)	75.9% (*n* = 22)	24.1% (*n* = 7)	
ICG‐FC^a^			
< 44 min (*n* = 30)	77.3% (*n* = 22)	26.7% (*n* = 8)	0.381
≥ 44 min (*n* = 42)	83.3% (*n* = 35)	16.7% (*n* = 7)	
Grade^b^			
I (*n* = 27)	88.9% (*n* = 24)	11.1% (*n* = 3)	0.143
II/III (*n* = 45)	73.3% (*n* = 33)	26.7% (*n* = 12)	
Max‐WBC^c^			
< 13 000 counts (*n* = 38)	92.1% (*n* = 35)	7.9% (*n* = 3)	0.008
≥ 13 000 counts (*n* = 34)	64.7% (*n* = 22)	35.3% (*n* = 12)	
Max‐CRP^d^			
< 13.98 mg/dL (*n* = 38)	89.5% (*n* = 34)	10.5% (*n* = 4)	0.040
≥ 13.98 mg/dL (*n* = 34)	67.6% (*n* = 23)	32.4% (*n* = 11)	
PTGBD			
− (*n* = 45)	80.0% (*n* = 36)	20.0% (*n* = 9)	> 0.999
+ (*n* = 27)	77.8% (*n* = 21)	22.2% (*n* = 6)	
EST/EPBD			
− (*n* = 40)	82.5% (*n* = 33)	17.5% (*n* = 7)	0.562
+ (*n* = 32)	75.0% (*n* = 24)	25.0% (*n* = 8)	
ENBD/EBS			
− (*n* = 51)	82.4% (*n* = 42)	17.6% (*n* = 9)	0.346
+ (*n* = 21)	71.4% (*n* = 15)	28.6% (*n* = 6)	

Abbreviations: EBS, endoscopic biliary stenting; ENBD, endoscopic nasobiliary drainage; EPBD, endoscopic papillary balloon dilatation; EST, endoscopic sphincterotomy; PTGBD, percutaneous transhepatic gallbladder drainage.

^a^ Interval between injection of ICG and start of fluorescent cholangiography.

^b^ Grade of acute cholecystitis.

^c^ The maximum of white blood cell counts between diagnosis of acute cholecystitis and surgery.

^d^ The maximum of C‐reactive protein between diagnosis of acute cholecystitis and surgery.

## 4 Discussion

According to TG‐18, it is recommended that surgery for patients with good status should be performed early in LC [1]. Moreover, DLC after recovery from AC was recommended for patients with poor status, which was classified according to the ASA‐PS and CCI [1, 2, 3, 4]. These recommendations were decided without articles regarding FC. After the publication of TG‐18, many articles comparing early LC and DLC for AC have been published, and their comparison remains controversial; however, those articles never included information on FC [5, 6, 7, 8, 9, 10, 11, 12, 13]. Recently, some articles regarding the clinical value of FC during early LC have been reported [30, 31, 32, 33, 34], but there have been no articles regarding the clinical value of FC during DLC. In this study, DLC with FC was safer than DLC without FC, with significantly lower intraoperative/postoperative complications, conversion rates, and blood loss. Among the patients with a potential status of urgent surgery, there were no intraoperative or postoperative complications during DLC with or without FC. Accordingly, DLC with FC is recognized as a safe procedure for patients with AC.

The most important role of FC is to visualize CD during LC. In this study, obesity affected visual acuity before dissection of Calot's triangle. This result was compatible with several previous reports [23, 34, 35, 36, 37, 38]; therefore, DLC with FC should be performed carefully in obese patients. Meanwhile, the preoperative biliary interventions (PTGBD, EST, RPBD, ENGBD, and/or EBS) did not affect the visuality of CD before dissection of Calot's triangle; therefore, DLC with FC can be performed safely regardless of preoperative biliary interventions. Another important feature of FC is CHANGE‐IN‐CD, which was affected by the values of Max‐WBC and/or Max‐CRP and was not apparent in one‐third of the patients with high values of Max‐WBC and/or Max‐CRP. These results are compatible with recent reports about the utilization of FC for AC, which reported that the detectabilities of biliary structures before and/or after dissection of Calot's triangle were affected by the degree of inflammation of the GB [30, 31, 32, 33, 34, 39, 40]. Accordingly, DLC with FC for patients with high levels of inflammatory markers between the diagnosis of AC and surgery should be performed carefully. Meanwhile, CHANGE‐IN‐CD was not affected by preoperative biliary interventions; therefore, DLC with FC can be performed safely regardless of preoperative biliary interventions.

As conventional bailout procedures, FDA and/or STC are well‐known procedures to avoid biliary injury during LC and were introduced in TG‐18 [29]. Recently, the clinical value of FC during FDA was reported [41], and our results illustrated that FDA with FC could be performed without intraoperative complications. Accordingly, visualization of biliary structures under the guidance of FC could facilitate FDA as easy and safe. FDA without FC had a high rate of intraoperative complications (25.0%); therefore, FDA with FC should be safer than that without FC. Despite no reports regarding STC with FC, because of visualization of CBD under FC, this procedure can be safely performed. In this study, there were no intraoperative complications after STC with or without FC. Accordingly, when DLC without FC is performed, STC might be safer than FDA.

As a distinctive bailout procedure with FC, PDCA or PDCD could be selected in patients with a detectable CD under FC before and/or during dissection of Calot's triangle. In this study, PDCA and PDCD with FC could be safely performed with an exact comprehension of the relationship between the biliary and arterial anatomies. Moreover, PDCA or PDCD without FC was recognized as the contrary concept to CVS [28]; however, the details of its reality have never been reported. According to our results of the high rate of intraoperative complications during PDCA without FC (30.8%), which was significantly different from that during PDCA with FC (*p* = 0.003), PDCA or PDCD without FC should be considered to encompass the risk of biliary and/or arterial injuries. Accordingly, when DLC without FC is performed, PDCA or PDCD should be carefully performed or possibly avoided, and conversion to OC should be considered.

This study has major limitations, with a limited sample size (*n* = 226) and no control arm. Despite the small number of studies, both the lack of intraoperative complications among the 144 patients who underwent DLC with FC and the high rate of intraoperative complications among the 82 patients who underwent DLC without FC (7.3%) were clarified; therefore, the safety of DLC with FC might be reliable. In addition, bailout procedures with FC had a greater advantage in terms of safety than those without FC.

In conclusion, routine utilization of FC is recommended for patients with AC who are scheduled for DLC. Among patients requiring bailout procedures during DLC, the utilization of FC has value in reducing intraoperative complications; therefore, DLC with FC is recognized as a safe procedure for patients with AC.

## Author Contributions

Study design: Tsuyoshi Igami. Data collection: Tsuyoshi Igami, Takuya Ishikawa, Kentaro Yamao, Yasuyuki Mizutani, Takashi Mizuno, Junpei Yamaguchi, Shunsuke Onoe, Masaki Sunagawa, Nobuyuki Watanabe, and Shoji Kawakatsu. Statistical analysis: Tsuyoshi Igami and Yukihiro Yokoyama. Draft writing: Tsuyoshi Igami. Draft revising: Tsuyoshi Igami, Hiroki Kawashima, and Tomoki Ebata.

## Conflicts of Interest

The authors declare no conflicts of interest.

## Data Availability

The data that support the findings of this study are available on request from the corresponding author. The data are not publicly available due to privacy or ethical restrictions.

## References

[1] K. Okamoto , T. Suzuki , T. Takada , et al., “Tokyo Guidelines 2018: Flowchart for the Management of Acute Cholecystitis,” Journal of Hepato‐Biliary‐Pancreatic Sciences 25 (2018): 55–72. 29045062 10.1002/jhbp.516

[2] ASA Physical Status Classification System , “Last Approved by the ASA House of Delegates on October 15, 2014,” https://www.asahq.org/resources/clinical‐information/asa‐physical‐status‐classification‐system.

[3] A. E. Abouleish , M. L. Leib , and N. H. Cohen , “ASA Provides Examples to Each ASA Physical Status Class,” ASA Monitor 79 (2015): 38–39.

[4] M. E. Charlson , P. Pompei , K. L. Ales , and C. R. MacKenzie , “A New Method of Classifying Prognostic Comorbidity in Longitudinal Studies: Development and Validation,” Journal of Chronic Diseases 40 (1987): 373–383. 3558716 10.1016/0021-9681(87)90171-8

[5] M. Barka , M. S. Jarrar , J. Sahli , Z. B. Abdessalem , F. Hamila , and S. Youssef , “Early Laparoscopic Cholecystectomy for Acute Cholecystitis: Should We Operate Beyond the First Week?,” Langenbeck's Archives of Surgery 408 (2023): 68, 10.1007/s00423-023-02816-5. 36701033

[6] A. Nassar , I. Elshahat , K. Forsyth , S. Shaikh , and M. Ghazanfar , “Outcome of Early Cholecystectomy Compared to Percutaneous Drainage of Gallbladder and Delayed Cholecystectomy for Patients With Acute Cholecystitis: Systematic Review and Meta‐Analysis,” HPB 24 (2022): 1622–1633. 35597717 10.1016/j.hpb.2022.04.010

[7] M. Abdalkoddus , J. Franklyn , R. Ibrahim , L. Yao , N. Zainudin , and S. Aroori , “Delayed Cholecystectomy Following Endoscopic Retrograde Cholangio‐Pancreatography Is Not Associated With Worse Surgical Outcomes,” Surgical Endoscopy 36 (2022): 2987–2993. 34231064 10.1007/s00464-021-08593-w PMC8259777

[8] G. Srikumar , M. J. McGuinness , N. Kau , C. Wells , and C. Harmston , “Cost Analysis of Index Versus Delayed Cholecystectomy for Scute Cholecystitis in a New Zealand Provincial Centre,” ANZ Journal of Surgery 92 (2022): 1675–1680. 35666130 10.1111/ans.17829

[9] J. Lucocq , P. Patil , and J. Scollay , “Acute Cholecystitis: Delayed Cholecystectomy Had Lesser Perioperative Morbidity Compared to Emergency Cholecystectomy,” Surgery 172 (2022): 16–22. 35461704 10.1016/j.surg.2022.03.024

[10] Y. N. Lin , Y. T. Wu , C. Y. Fu , et al., “Evaluating the Advantages of Treating Acute Cholecystitis by Following Tokyo Guidelines 2018 (TG18): A Study Emphasizing Clinical Outcomes and Medical Expenditures,” Surgical Endoscopy 35 (2021): 6623–6632. 33258028 10.1007/s00464-020-08162-7

[11] M. Di Martino , I. Mora‐Guzman , V. V. Jodra , et al., “Laparoscopic Cholecystectomy for Acute Cholecystitis: Is the Surgery Still Safe Beyond the 7‐Day Barrier? A Multicentric Observational Study,” Updates in Surgery 73 (2021): 261–272. 33211289 10.1007/s13304-020-00924-1

[12] A. Discolo , S. Reiter , B. French , et al., “Outcomes Following Early Versus Delayed Cholecystectomy Performed for Scute Cholecystitis,” Surgical Endoscopy 34 (2020): 3204–3210. 31482348 10.1007/s00464-019-07095-0

[13] A. Kohga , K. Suzuki , T. Okumura , et al., “Outcomes of Early Versus Delayed Laparoscopic Cholecystectomy for Acute Cholecystitis Performed at a Single Institution,” Asian Journal of Endoscopic Surgery 12 (2019): 74–80. 29611896 10.1111/ases.12487

[14] M. Yokoe , J. Hata , T. Takada , et al., “Tokyo Guidelines 2018: Diagnostic Criteria and Severity Grading of Acute Cholecystitis (With Videos),” Journal of Hepato‐Biliary‐Pancreatic Sciences 25 (2018): 41–54. 29032636 10.1002/jhbp.515

[15] H. Gomi , J. S. Solomkin , D. Schlossberg , et al., “Tokyo Guidelines 2018: Antimicrobial Terapy for Acute Cholangitis and Cholecystitis,” Journal of Hepato‐Biliary‐Pancreatic Sciences 25 (2018): 3–16. 29090866 10.1002/jhbp.518

[16] S. Kiriyama , K. Kozaka , T. Takada , et al., “Tokyo Guidelines 2018: Diagnostic Criteria and Severity Grading of Acute Cholangitis (With Videos),” Journal of Hepato‐Biliary‐Pancreatic Sciences 25 (2018): 17–30. 29032610 10.1002/jhbp.512

[17] F. Miura , K. Okamoto , T. Takada , et al., “Tokyo Guidelines 2018: Initial Management of Acute Biliary Infection and Flowchart for Acute Cholangitis,” Journal of Hepato‐Biliary‐Pancreatic Sciences 25 (2018): 31–40. 28941329 10.1002/jhbp.509

[18] S. Mukai , T. Itoi , T. H. Baron , et al., “Indication and Techniques of Biliary Drainage for Acute Cholangitis in Updated Tokyo Guidelines 2018,” Journal of Hepato‐Biliary‐Pancreatic Sciences 24 (2017): 537–549. 28834389 10.1002/jhbp.496

[19] Y. Mori , T. Itoi , T. H. Baron , et al., “Tokyo Guidelines 2018: Management Strategies for Gallbladder Drainage in Patients With Acute Cholecystitis (With Videos),” Journal of Hepato‐Biliary‐Pancreatic Sciences 25 (2018): 87–95. 28888080 10.1002/jhbp.504

[20] T. Igami , H. Usui , T. Ebata , et al., “Single‐Incision Laparoscopic Cholecystectomy for Porcelain Gallbladder: A Case Report,” Asian Journal of Endoscopic Surgery 6 (2013): 52–54. 23347708 10.1111/ases.12008

[21] T. Igami , T. Ebata , Y. Yokoyama , et al., “Single‐Incision Laparoscopic Cholecystectomy After Endoscopic Nasogallbladder Drainage: A Case Report,” Medical Principles and Practice 24 (2015): 496–499. 26022235 10.1159/000430951 PMC5588253

[22] T. Igami , T. Aoba , T. Ebata , Y. Yokoyama , G. Sugawara , and M. Nagino , “Single‐Incision Laparoscopic Cholecystectomy for Cholecystitis Requiring Percutaneous Transhepatic Gallbladder Drainage,” Surgery Today 45 (2015): 305–309. 25139210 10.1007/s00595-014-1003-4

[23] T. Igami , M. Nojiri , K. Shinohara , et al., “Clinical Value and Pitfalls of Fluorescent Cholangiography During Single‐Incision Laparoscopic Cholecystectomy,” Surgery Today 46 (2016): 1443–1450. 27002714 10.1007/s00595-016-1330-8

[24] M. Nojiri , T. Igani , H. Tanaka , et al., “Application of Fluorescent Cholangiography for Determination of the Resection Line During a Single‐Incision Laparoscopic Cholecystectomy for a Benign Lesion of the Cystic Duct: Preliminary Experience,” Surgical Laparoscopy, Endoscopy & Percutaneous Techniques 26 (2016): e171–e173. 10.1097/SLE.0000000000000342 27846167

[25] M. Nojiri , T. Igami , Y. Toyoda , et al., “Application of Fluorescent Cholangiography During Single‐Incision Laparoscopic Cholecystectomy for Cholecystitis With a Right‐Sided Round Ligament: Preliminary Experience,” Journal of Minimal Access Surgery 14 (2018): 244–246. 29226884 10.4103/jmas.JMAS_159_17 PMC6001308

[26] Y. Asai , T. Igami , T. Ebata , et al., “Application of Fluorescent Cholangiography During Single‐Incision Laparoscopic Cholecystectomy in the Cystohepatic Duct Without Preoperative Diagnosis,” ANZ Journal of Surgery 91 (2021): 470–472. 32681758 10.1111/ans.16162

[27] T. Igami , Y. Asai , T. Minamai , et al., “Clinical Value of Fluorescent Cholangiography for Th Infraportal Type of Right Posterior Bile Duct,” Minimally Invasive Therapy & Allied Technologies 32 (2023): 256–263. 37288773 10.1080/13645706.2023.2217915

[28] S. M. Strasberg , M. Hertl , and N. J. Soper , “An Analysis of the Problem of Biliary Injury During Laparoscopic Cholecystectomy,” Journal of the American College of Surgeons 180 (1995): 101–125. 8000648

[29] G. Wakabayashi , Y. Iwashita , T. Hibi , et al., “Tokyo Guidelines 2018: Surgical Management of Acute Cholecystitis: Safe Steps in Laparoscopic Cholecystectomy for Acute Cholecystitis (With Videos),” Journal of Hepato‐Biliary‐Pancreatic Sciences 25 (2018): 73–86. 29095575 10.1002/jhbp.517

[30] G. Piccolo , M. Barabino , F. Lecchi , et al., “Utility of Near Infrared Fluorescent Cholangiography in Detecting Biliary Structures During Challenging Minimally Invasive Cholecystectomy,” Langenbeck's Archives of Surgery 408 (2023): 282, 10.1007/s00423-023-02995-1. 37462733

[31] S. Symeonidis , I. Mantzoros , E. Anestiadou , et al., “Biliary Anatomy Visualization and Surgeon Satisfaction Using Standard Cholangiography Versus Indocyanine Green Fluorescent Cholangiography During Elective Laparoscopic Cholecystectomy: A Randomized Controlled Trial,” Journal of Clinical Medicine 13 (2024): 864, 10.3390/jcm13030864. 38337557 PMC10856121

[32] F. Di Maggio , N. Hossain , A. De Zanna , et al., “Near‐Infrared Fluorescent Cholangiography Can Be a Usuful Adjunct During Emergency Cholecystectomies,” Surgical Innovation 29 (2020): 526–531. 32936054 10.1177/1553350620958562

[33] D. Daskalaki , E. Fernandes , X. Wang , et al., “Indocyanine Green (ICG) Fluorescent Cholangiography During Robotic Cholecystectomy: Results of 184 Concecutive Cases in a Single Institution,” Surgical Innovation 21 (2014): 615–621. 24616013 10.1177/1553350614524839

[34] F. Dip , E. LoMenzo , L. Satotto , et al., “Randomized Trial of Near‐Infrared Incisionless Fluorescent Cholangiography,” Annals of Surgery 270 (2019): 992–999. 30614881 10.1097/SLA.0000000000003178

[35] N. C. Buchs , F. Pugin , D. E. Azagury , et al., “Real‐Time Near‐Infrared Fluorescent Cholangiography Could Shorten Operative Time During Robotic Single‐Site Cholecystectomy,” Surgical Endoscopy 27 (2013): 3897–3901. 23670747 10.1007/s00464-013-3005-5

[36] T. Aoki , M. Murakami , D. Yasuda , et al., “Untraoperative Fluorescent Imaging Using Indocyanine Green for Liver Mapping and Cholangiography,” Journal of Hepato‐Biliary‐Pancreatic Sciences 17 (2010): 590–594. 19844652 10.1007/s00534-009-0197-0

[37] S. L. Vlek , D. A. van Dan , S. M. Rubinstein , et al., “Biliary Tract Visualization Using Near‐Infrared Imaging With Indocyanine Green During Laparoscopic Cholecystectomy: Results of a Systematic Review,” Surgical Endoscopy 31 (2017): 2731–2742. 27844236 10.1007/s00464-016-5318-7 PMC5487840

[38] V. Pax , S. Schneider‐Koriath , M. Schoiz , et al., “Fluorescent Cholangiography in Comparison to Radiographic Cholangiography During Laparoscopic Cholecystectomy,” Zentralblatt für Chirurgie 143 (2018): 35–41. 29166696 10.1055/s-0043-117495

[39] A. Pesce , G. Piccolo , G. La Greca , et al., “Utility of Fluorescent Cholangiography During Laparoscopic Cholecystectomy: A Systematic Review,” World Journal of Gastroenterology 21 (2015): 7877–7883. 26167088 10.3748/wjg.v21.i25.7877 PMC4491975

[40] Y. Agnus , A. Pesce , L. Bonl , et al., “Fluorescence‐Based Cholangiography: Preliminary Results From the IHU‐IRCAD‐EAES EURO‐FIGS Registry,” Surgical Endoscopy 34 (2020): 3888–3896. 31591654 10.1007/s00464-019-07157-3

[41] I. C. Lee and J. Li , “Indocyanine‐Green Fluoresced Imaging Guided Fundus‐First Approach for Early Laparoscopic Cholecystectomy in a Patient With Acute Pancreatitis and a Difficult Gallbladder,” Asian Journal of Surgery 47 (2024): 626–627. 37805333 10.1016/j.asjsur.2023.09.140

